# Supplementary material for: Loss of LCMT1 and biased protein phosphatase 2A heterotrimerization drive prostate cancer progression and therapy resistance
Source: Nat Commun. 2023 Aug 29;14:5253. doi: 10.1038/s41467-023-40760-6 (PMC10465527; doi:10.1038/s41467-023-40760-6)
Supplement: Supplementary file 1 — Supplementary Information [file 41467_2023_40760_MOESM1_ESM.pdf]

Supplementary Figure 1

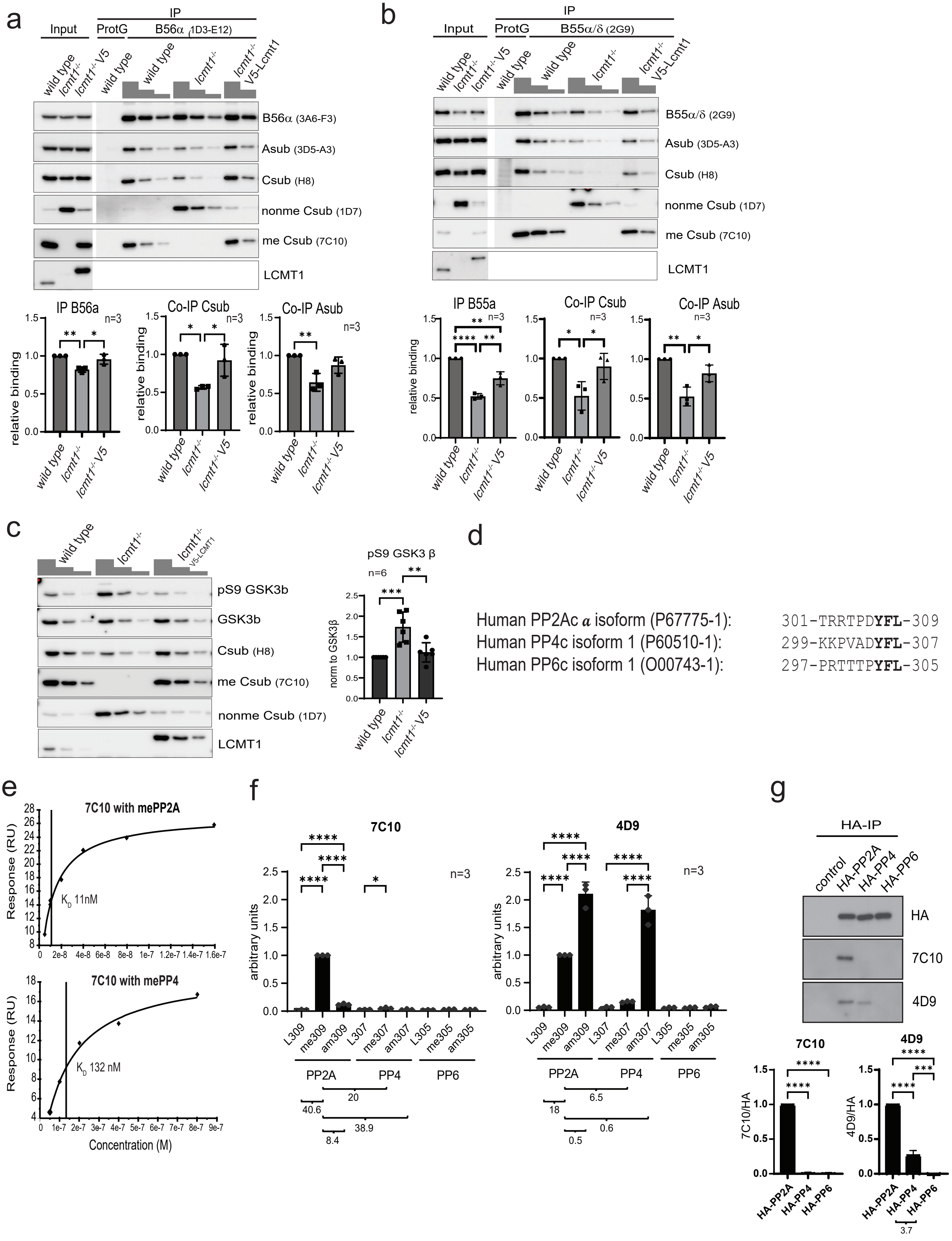

Supplementary Figure 1:

a, Reduced levels of B56 subunit–containing holoenzymes in cells lacking PP2A methylation can be restored by reexpressing V5-Lcmt1. Immunoblotting of lysates and anti-B56α immunoprecipitates from lysates of HAP1 wild type, lcmt1-/-, and lcmt1-/- cells expressing V5-Lcmt1. 1/10 of the input lysate was loaded for the B56α blot, and 1/160 of the input lysate for all other blots. The panels originate from 3 independent blotting membranes, which were sequentially incubated with the indicated antibodies. The blots are representative of n=3 independent immunoprecipitation experiments. The amounts of immunoprecipitated B56α was quantified, and the coimmunoprecipitated A and C subunit levels were normalized to the B56α levels, which were set to 1 for the wild-type cells. Statistical significance of quantified proteins levels was assessed using one way ANOVA followed by Tukey's HSD (one-sided) as a post hoc test. B56 IP: ** P =0.0049, * P =0.018, CoIP Csub: * P =0.0114 * P =0.0270 (from left to right), CoIP Asub: ** P= 0.0079. Data are presented as mean values ± s.d. **b,** Reduced levels of B55 subunit–containing holoenzymes in cells lacking PP2A methylation can be restored by reexpressing V5-Lcmt1. Immunoblotting of lysates and anti-B55α/δ immunoprecipitates from lysates of HAP1 wildtype and Lcmt1-/-, and Lcmt1-/- cells expressing V5-Lcmt1. 1/160 of the input was loaded on blots. The panels originate from 3 independent blotting membranes, which were sequentially incubated with the indicated antibodies. The blots are representative of n=3 independent immunoprecipitation experiments. The amounts of immunoprecipitated B55α/δ was quantified, and the coimmunoprecipitated A and C subunit levels were normalized to the B55α/δ levels, which were set to 1 for the wildtype cells. Statistical significance of quantified protein levels was assessed using one way ANOVA followed by Tukey's HSD (one-sided) as a post hoc test. B55 IP **** P <0.0001, ** P= 0.0024 ** P= 0.0040, CoIP Csub * P= 0.0148, * P= 0.0405, CoIP Asub ** P= 0.0018, * P = 0.0185 (from left to right). Data are presented as mean values ± s.d. **c,** Immunoblotting of lysates of HAP1 wildtype, lcmt1-/- cells, and lcmt1-/- cells stably expressing V5-Lcmt1 using indicated antibodies. The blots are representative of n=6 experiments. The GSK3beta and pS9 GSK3beta signals were quantified and normalized to GSK3beta, and the wildtype was set to 1. The statistical significance of immunoblotting was assessed using an un-paired two-sided Student's t-test. *** P= 0.0004 P=**0.0020. Data are presented as mean values ± s.d. **d,** Alignment of the C termini of mammalian catalytic PP2A-C, PP4-C and PP6-C subunits. **e,** Monoclonal antibody 7C10-C5 is highly specific for the carboxymethylated catalytic subunit of protein phosphatase 2A (PP2A-C). The affinity of 7C10-C5 to peptides mePP2A (ac-HVTRRTPDYFL-CH3) and mePP4 (ac-PSKKPVADYFL-CH3) was analyzed using Surface Plasmon Resonance Spectrometer Biacore. 7C10-C5 displays high specificity for methylated PP2A-C with a K_D of 11nM, an affinity that was 12-fold higher than that for methylated PP4-C with a K_D of 132nM. **f,** We also compared the properties of 7C10-C5 to the monoclonal antibody 4D9 that was generated against a PP2A-C carboxy-terminal peptide with an amidated carboxyl-group at Leu 309, with the rationale being to mimic the charge neutralization of the naturally occurring α–carboxymethylation. An ELISA plate was coated with peptides L309 (ac-HVTRRTPDYFL), meL309 (ac-HVTRRTPDYFL-CH3), amL309 (ac-HVTRRTPDYFL-NH2), L307 (ac- PSKKPVADYFL), meL307 (ac-PSKKPVADYFL-CH3), amL307 (ac-PSKKPVADYFL-NH2), L305 (ac-IPPRTTTPYFL), meL305 (ac-IPPRTTTPYFL-CH3) or amL305 (ac-IPPRTTTPYFL-NH2) at 2 µg/ml in TBS and incubated with purified 7C10 or purified 4D9 (1µg/ml). Values were normalized to meL309, which was arbitrarily set to 1. Average and standard deviation of n=3 experiments are shown. ELISA revealed a preference of 4D9 for the amidated PP2A-C as well as PP4-C C-terminal peptides that were recognized with at least 2-fold

higher strength than the methylated PP2A-C peptide. 7C10-C5 on the other hand, displayed a clear preference with >6-fold higher signals for methylated vs. amidated PP2A-C and no binding seen to the amidated form of PP4-C. The statistical significance was assessed using one way ANOVA followed by Tukey's HSD (one-sided) as a post hoc test. 7C10: **** P >0.0001 * P =0.0447, 4D9: **** P >0.0001. **g**, The high specificity of 7C10-C5 for PP2A-C and substantial cross-reactivity of 4D9 with PP4-C. Immunoblotting of anti-HA immunoprecipitates from lysates of NIH3T3 cells either infected with retroviral supernatants of pBabe hygro or pBabe hygro HA-PP2A-C, pBabe hygro HA-PP4-C or pBabe hygro HA PP6-C using the indicated antibodies. To equilibrate the HA immunoprecipitates levels 2 times more of the HA-PP4 and 3 times more of the HA-PP6-C immunoprecipitates were loaded compared to the HA-PP2A. The panels originate from 3 different blotting membranes, each incubated with the indicated antibody. The blots are representative of n=3 independent immunoprecipitation experiments. The statistical significance was assessed using one way ANOVA followed by Tukey's HSD (one-sided) as a post hoc test 7C10 **** P >0.0001, 4D9 *** P= 0.0005. Data are presented as mean values ± s.d.

Supplementary Figure 2

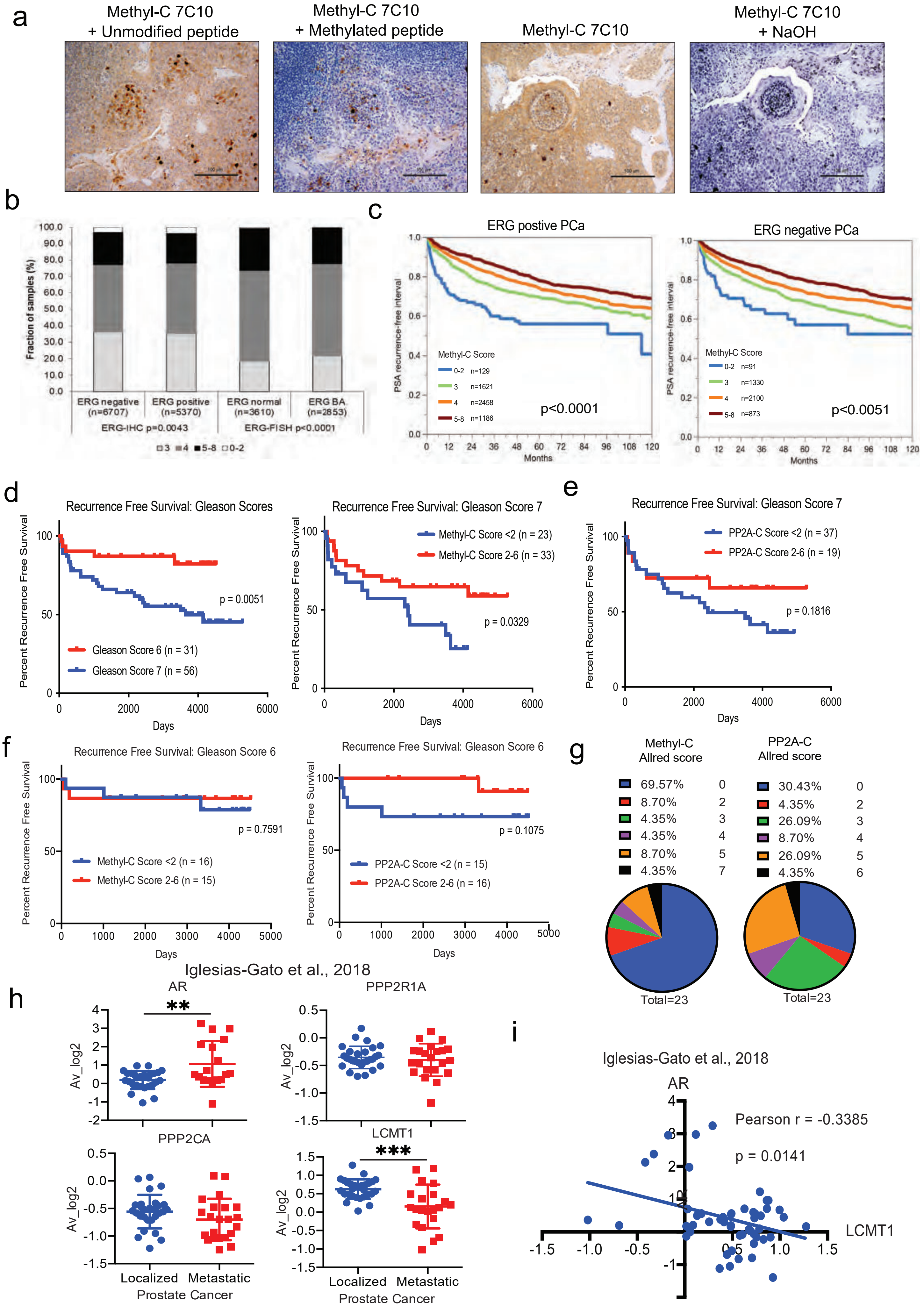

Supplementary Figure 2: Methyl-PP2A-C loss is associated with prostate cancer progression
a, Specificity of methyl-PP2A-C antibody 7C10-C5. Peptide competition with unmethylated or α-carboxymethylated peptides confirmed the high specificity of the 7C10-C5 antibody. Incubation with NaOH resulted in abolished methylation and loss of methyl-C signal. The experiment was repeated at least twice independently with similar observations. **b, and c,** No association between low immunohistochemistry methyl-C scores and presence of the TMPRSS2::ERG gene fusion in prostate cancer. ERG fusion was analyzed by immunohistochemistry (ERG-IHC) and by fluorescence in-situ hybridization (ERG-FISH) using an ERG break-apart probe. For b and c Chi square test and Log-rank test were used to draw the statistics respectively as shown in the figures. **d, e,** and **f,** Recurrence free survival in Gleason 6 vs 7 disease and association of methyl-C score with survival in Gleason 7 prostate cancer. Allred scoring system was used to score the staining. The Allred score combines the percentage of positive cells and the intensity of the staining. Survival curves compared by Log-rank (Mantel Cox) Test, p-values shown. **g,** Distribution of methyl-C and PP2A-C staining in prostate cancer tissue microarray in localized and metastatic prostate cancer samples. **h,** System wide quantitative proteomic analysis of localized ($n = 28$) and metastatic ($n = 22$) prostate tumors for the indicated proteins (Iglesias-Gato et al., 2018). Error bars mean ± s.d., Students two-tailed t-test, * indicates p-value: 0.0020 (AR), p-value: 0.5194 (PPP2R1A), p-value: 0.1484 (PPP2CA), p-value: 0.0006 (LCMT1). **i,** Correlation between AR and LCMT1 protein expression, Pearson's correlation coefficient, two-tailed.

Supplementary Table 1

	No. of patients (%)	
	**Study cohort on TMA (n=17,747)**	**Biochemical relapse among categories**
**Follow-up (mo)**		
n	14464 (81.5%)	3612 (25%)
Mean	56.3	-
Median	48	-
Age (y)		
≤50	433 (2.4%)	66 (15.2%)
51-59	4341 (24.5%)	839 (19.3%)
60-69	9977 (56.4%)	2073 (20.8%)
≥70	2936 (16.6%)	634 (21.6%)
**Pretreatment PSA (ng/ml)**		
<4	2225 (12.6%)	313 (14.1%)
4-10	10520 (59.6%)	1696 (16.1%)
10-20	3662 (20.8%)	1043 (28.5%)
>20	1231 (7%)	545 (44.3%)
**pT stage (AJCC 2002)**		
pT2	11518 (65.2%)	1212 (10.5%)
pT3a	3842 (21.7%)	1121 (29.2%)
pT3b	2233 (12.6%)	1213 (54.3%)
pT4	85 (0.5%)	63 (74.1%)
**Gleason grade**		
≤3+3	3570 (20.3%)	264 (7.4%)
3+4	9336 (53%)	1436 (15.4%)
3+4 Tert.5	798 (4.5%)	165 (20.7%)
4+3	1733 (9.8%)	683 (39.4%)
4+3 Tert.5	1187 (6.7%)	487 (41%)
≥4+4	999 (5.7%)	531 (53.2%)
**pN stage**		
pN0	10636 (89.4%)	2243 (21.1%)
pN+	1255 (10.6%)	700 (55.8%)
**Surgical margin**		
Negative	14297 (80.8%)	2307 (16.1%)
Positive	3388 (19.2%)	1304 (38.5%)

NOTE: Numbers do not always add up to 17,747 in the different categories because of cases with missing data. Abbreviation: AJCC, American Joint Committee on Cancer.

Supplementary Table 1

Summary of the clinical and pathological characteristics of the patient cohort represented by the 17,747 samples tissue microarray.

Supplementary Table 2

		n evaluable	0-2 (%)	3 (%)	4 (%)	5-8 (%)	p value
**all cancers**		16149	3.3	34.9	42	19.7	
**Tumor stage**	pT2	10401	2.7	33.4	42.7	21.2	<0.0001
	pT3a	3520	3.9	36.7	41.4	17.9	
	pT3b-4	2162	5.3	39.5	39.8	15.4	
**Gleason grade**	≤3+3	3172	1.3	27.6	43.2	27.9	<0.0001
	3+4	8501	2.7	33.5	43.6	20.2	
	3+4 Tert.5	738	6	43.4	38.2	12.5	
	4+3	1564	4.5	37	42.3	16.2	
	4+3 Tert.5	1104	6.8	45.7	37.1	10.3	
	≥4+4	913	7.1	42.5	37.6	12.8	
**quantitative Gleason**	3+4 ≤5%	2190	2.4	29.4	47.4	20.9	<0.0001
	3+4 6-10%	2110	3.1	29.4	45.9	21.6	
	3+4 11-20%	1821	2.5	34.4	42.4	20.6	
	3+4 21-30%	946	2.1	37.1	41.9	18.9	
	3+4 31-49%	788	3	35.7	42.3	19	
	4+3 50-60%	622	6	43.4	38.2	12.5	
	4+3 61-80%	566	3.5	34.6	45.2	16.7	
	4+3 >80%	139	4.8	38.5	41.9	14.8	
**Lymph node metastasis**	N0	9636	3.9	37.4	41.2	17.5	<0.0001
	N+	1192	6	45.6	37	11.4	
**Preop. PSA level (ng/ml)**	<4	2017	3.4	31.1	42.6	22.9	<0.0001
	4-10	1152	4.9	39.9	39.3	15.9	
	11-20	9524	2.9	34.3	42.6	20.2	
	>20	3355	3.9	37.5	40.9	17.7	
**Surgical margin**	negative	12944	3.2	34.4	42.2	20.3	<0.0001
	positive	3147	3.8	37.5	41.3	17.4	

Supplementary Table 2
Association between histo-pathological parameters and the methyl-C score in the tissue microarray cohort of 16,149 prostate cancers that were interpretable for methyl-C immunohistochemistry. Chi square test was used to calculate the statistics.

Supplementary Figure 3

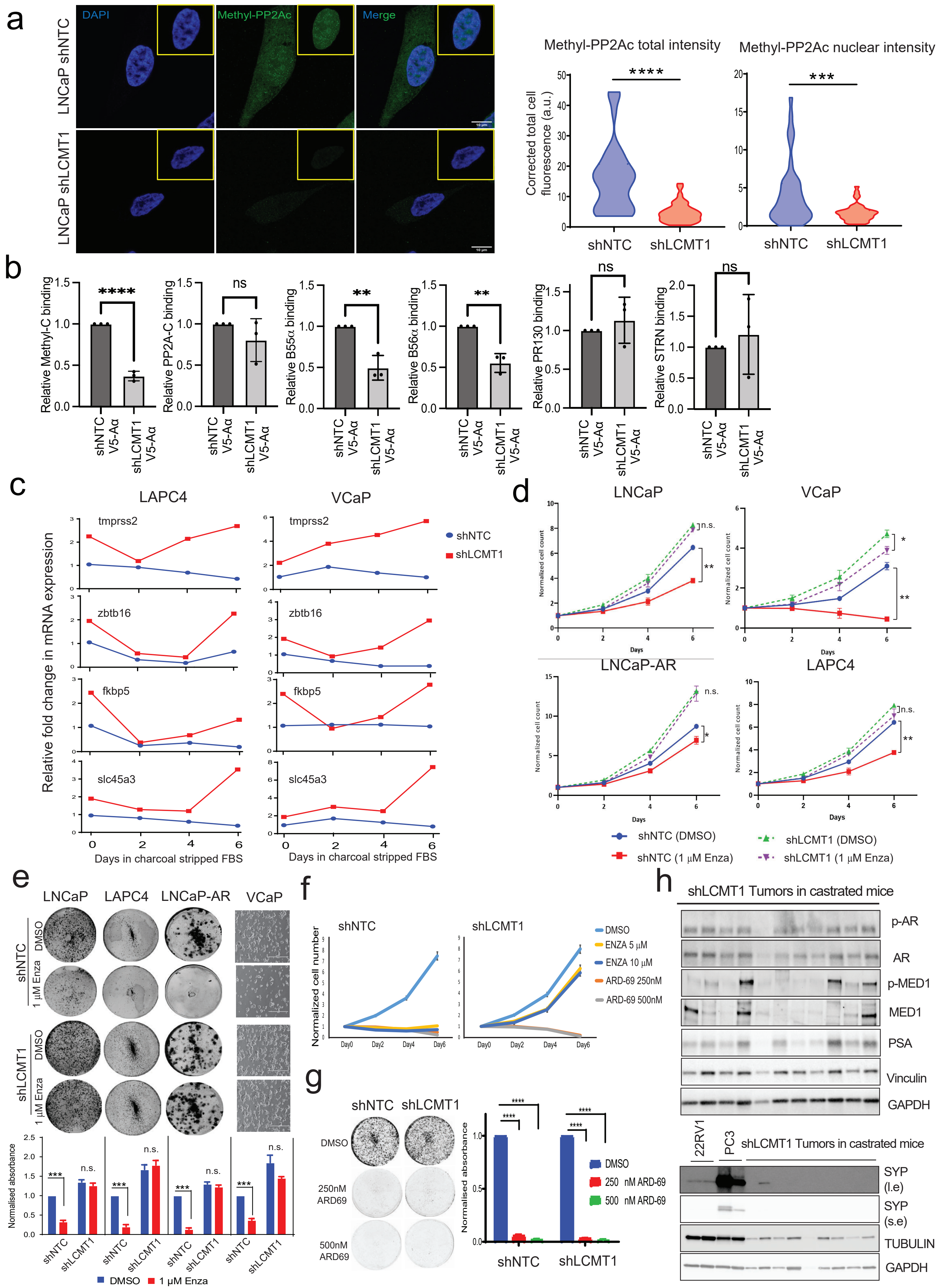

Supplementary Figure 3: Reduced methyl-C and PP2A holoenzyme in LCMT1 knockdown cells. a, LCMT1 knock-down results in reduced methyl-C levels. Representative confocal microscopy images show the reduced levels of nuclear and total methyl-C in LCMT1 knock-down LNCaP cells (left). Quantification of total and nuclear intensity of methyl-C is shown (right) in the form of corrected total cell fluorescence. Scale bar: 10μm. The p values were calculated using two tailed t-tests. From left to right, n = 40, 40, 41, and 41 cells counted. *p < 0.001, **p < 0.0001. **b,** Quantification of immunoblots from Figure 2B showing the relative presence of the indicated PP2A subunits in the holoenzyme upon LCMT1 loss, error bars mean ± s.d., n=3 biologically independent experiments. Student's two-tailed t-test, p-value: <0.0001 (Methyl-C), 0.2598 (PP2A-C), 0.0043 (B55α), 0.0025 (B56α), 0.4819 (PR130), 0.6060 (STRN). **c**, LCMT1 loss benefits PCa cells with sustained AR transcriptional activity under androgen deprivation. qRT-PCR for AR-regulated genes in cells grown in CSS-containing medium. **d,** Resistance to anti-androgen in LCMT1 silenced prostate cancer cells. Proliferation assay showing increased growth and resistance to second-generation anti-androgen enzalutamide by cells stably expressing shLCMT1 compared to shNTC controls. The error bar represents the mean ± s.d. (n = 3). Statistical significance as calculated by t-test is represented as *p < 0.05, **p < 0.01. **e,** Colony formation assay in the presence of enzalutamide. Cells were cultured in the presence of vehicle, or 1μM enzalutamide for 14days followed by staining (top) and quantification (bottom). The error bar represents the mean ± s.d. among the triplicates. Statistical significance as calculated by t-test is represented as ***p < 0.001, ns=non-significant. **f,** LCMT1 silenced cells are sensitive to AR degradation. Proliferation assay showing sensitivity and resistance of shLCMT1-LNCaP cells to ARD-69 (AR PROTAC) and enzalutamide, respectively, compared to DMSO controls. The error bar represents the mean ± s.d. (n = 3). **g,** Colony formation assay demonstrating the effect of ARD-69 in shLCMT1- and shNTC- LNCaP cells. Cells were cultured in the presence of vehicle or 1 μM ARD-69 for 12-14days, followed by staining (left) and quantification (right). The error bar represents the mean ± s.d. (n = 3). Statistical significance is represented as ****p < 0.0001. **h,** Active AR signaling in castration-resistant tumors (n = 11) derived from LCMT1 silenced LNCaP cells. Individual tumor lysates (Figure 2g) were immunoblotted for the indicated proteins..

Supplementary Figure 4

a

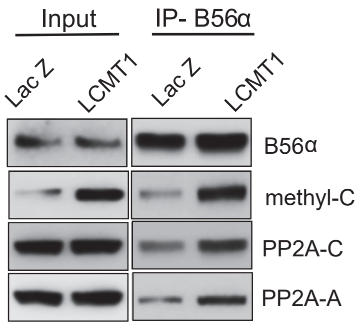

b

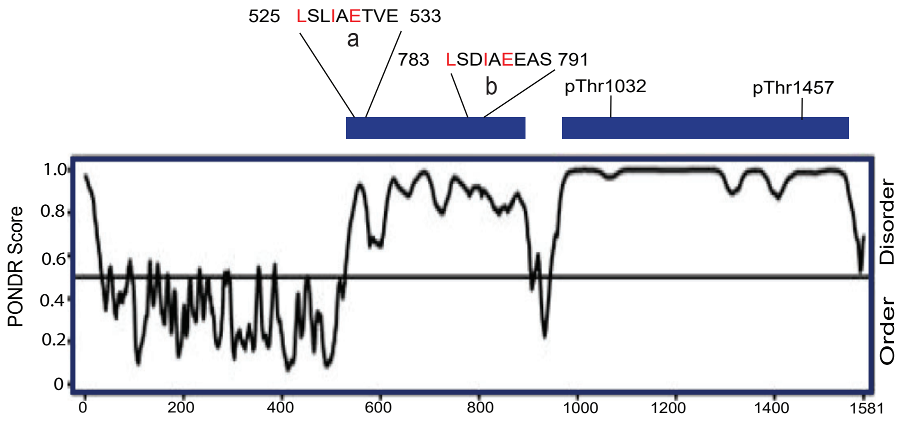

c

i)

	Target	# B56 docking motif	Consensus LxxIxE	Consensus Similarity significance	Normalized Spep	Disorder score
Validated	BUB1	2	LSPIIEDSR, CLQIPEKLE	*<0.0001	0.75, 0.48	0.44, 0.38
Validated	CDCA2	2	LSPIPELPE, LPEVPEMTP	*<0.0001	0.80, 0.54	0.64, 0.65
Novel	MED1	2	LSLIAETVE, LSDIAEEAS	*<0.0001	0.47, 0.61	0.43, 0.70

ii)

Conservation of B56α binding motif on MED1 across species

```
             a          LxxIxE                          b             LxxIxE
Human    TIQADTPALSLIAETVEDMVKKNLPPASSPGY    DSIGPDVTDILSDIAEEASKLPSTSDDCPAIG
Mouse    TIQADTPALSLIAETVEDMVKKNLPPASSPGY    DSIGPDVTDILSDIAEEASKLPSTSDDCPPIG
Rat      TIQADTPALSLIAETVEDMVKKNLPPASSPGY    DSIGPDVTDILSDIAEEASKLPSTSDDCPPIG
Frog     TIQADTPALSLIAETVEDMVKKNLPPASSPGY    DAIIPDVTDILSDIAEEASKLPGPGEDCPNLG
Chicken  TIQADTPALSLIAETVEDMVKKNLPPASSPGY    DSIGADVTDILSDIAEEASKLPATNEDCPPIG
Cattle   TIQADTPALSLIAETVEDMVKKNLPPASSPGY    DSIGPDVTDILSDIAEEASKLPSTSDDCPPIG
Horse    TIQADTPALSLIAETVEDMVKKNLPPASSPGY    DSIGPDVTDILSDIAEEASKLPSTSDDCPPIG
Boar     TIQADTPALSLIAETVEDMVKKNLPPASSPGY    DSIGPDVTDILSDIAEEASKLPSTSDDCPPIG
Dog      TIQADTPALSLIAETVEDMVKKNLPPASSPGY    DSIGPDVTDILSDIAEEASKLPSTNDDCPPIG
Hamster  TIQADTPALSLIAETVEDMVKKNLPPASSPGY    DSIGPDVTDILSDIAEEASKLPSTNDDCPPIG
```

d

HEK293-T nuclear lysate

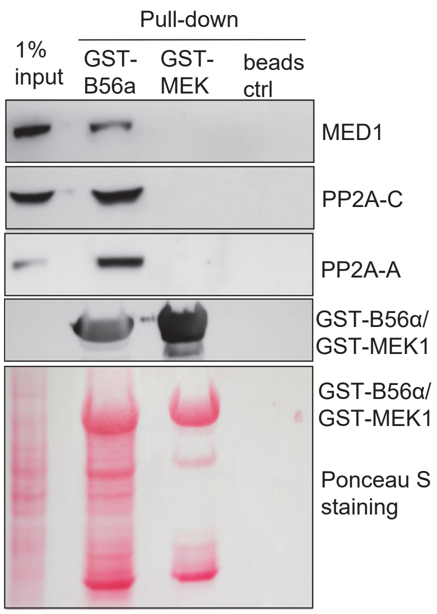

Supplementary Figure 4: PP2A-B56α stabilization by LCMT1 targets MED1-AR for dephosphorylation. **a,** Stabilized AB56αCme heterotrimer complex in LNCaP cells over-expressing LCMT1. Co-immunoprecipitation analysis using lysates from stably over-expressing LCMT1- and LacZ-LNCaP cells with the ProtG B56α (1D3-E12) antibody, demonstrating increased stabilization between PP2A-Aα, methylated-PP2AC (Methyl-C), B56α heterotrimer complex. 5% input was used. **b,** Graph showing the intrinsic disorder region (IDR) of MED1 as calculated by the VSL2 algorithm (http://www.pondr.com/). The two B56α binding SLiM motif and the two known threonine phosphorylation sites are indicated above the disorder score graph. **c,** i- Comparison of the consensus B56 α motif on MED1 to well established PP2A-B56α substrates BUB1 and CDCA2. ii- Conservation of the two B56α sequence motifs on MED1 across species. The LxxIxE motif is highlighted in yellow. **d,** Purified B56α binds to endogenous MED1. GST-pulldown assay with purified GST-B56α and nuclear lysates from HEK293-T cells. GST-MEK1 was used as a control. For **a** and **d,** the results shown are representative of three independent biological replicates.

Supplementary Figure 5

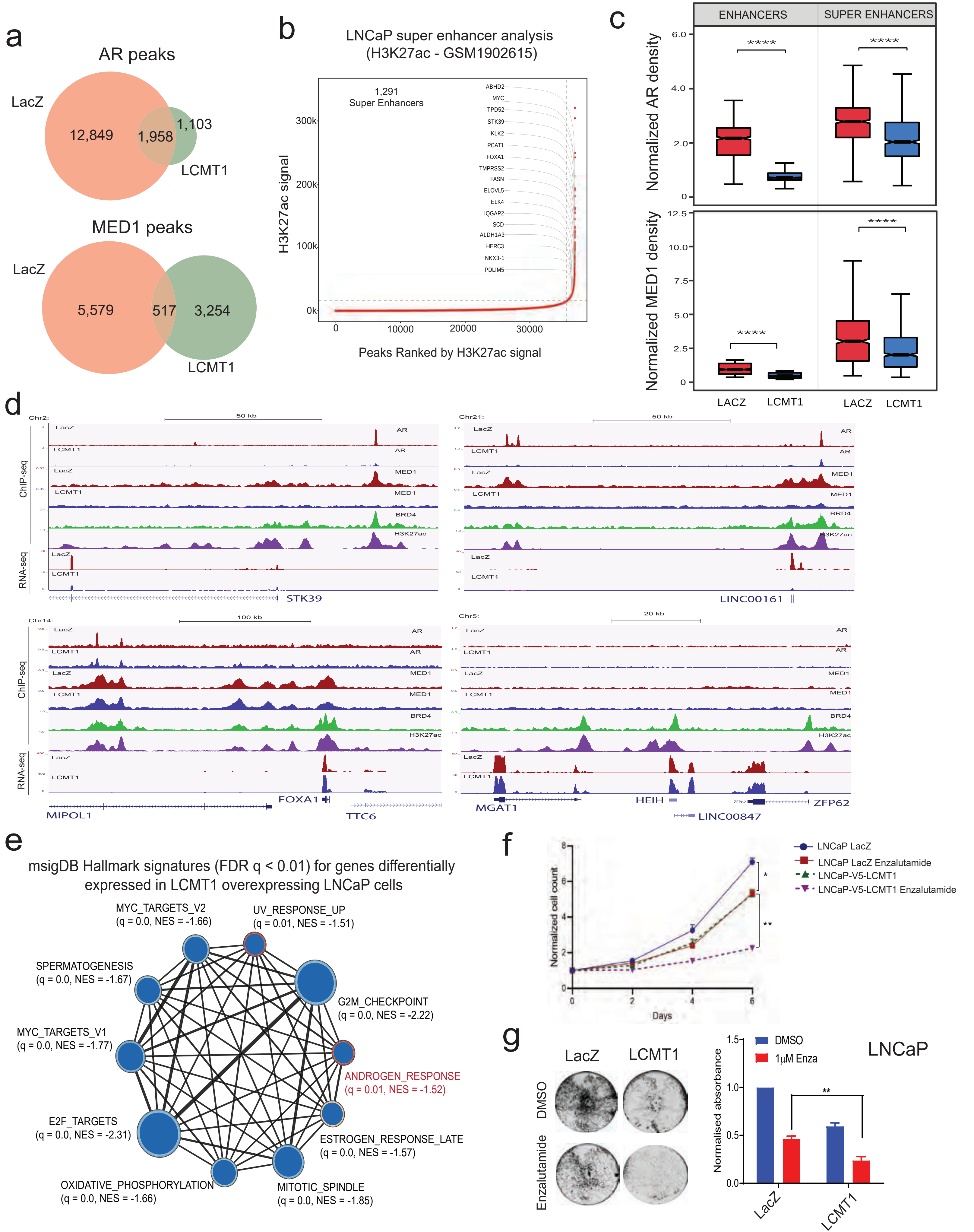

Supplementary Figure 5: LCMT1 antagonizes AR transcriptional program and anti-androgen resistance. a, Venn diagram illustrating the number of AR- and MED1-enriched regions in LacZ and LCMT1 overexpressing LNCaP cells. **b,** Rank-ordered H3K27ac ChIP-seq signal showing the presence of enhancers and SE regions in LNCaP cells. **c,** LCMT1 overexpression leads to the eviction of AR and MED1 from the chromatin. Boxplots of normalized AR and MED1 enrichment (reads per million mapped) at the enhancers and super-enhancers. p values calculated from Mann-Whitney u-tests are shown (****$p < 0.0001$). The middle line shows the median, the box limits show the 75th and 25th percentiles and the whiskers show the minimum and maximum values. **d,** Genome browser tracks of AR and MED1 binding at AR/MED1 regulated and non-regulated loci in the indicated cells, and BRD4/H3K27ac tracks from LNCaP cells. The tracks at the bottom show the RNA-seq gene expression. **e,** GSEA network plot showing the reversal of the msigDB Hallmark Androgen response and MYC target signature in LNCaP-LCMT1 cells compared to LacZ control cells, as determined by GSEA with FDR $q < 0.1$. **f,** Proliferation assay displaying the suppressed growth and increased sensitivity to enzalutamide (1μM) of LNCaP-V5-LCMT1 cells compared to parental control cells. **g,** LNCaP-V5-LCMT1 colony formation assay in the presence of 1μM enzalutamide. The quantification of the colonies showing decreased proliferation and increased sensitivity of LNCaP-V5-LCMT1 cells to enzalutamide is shown on the right. For **f** and **g**, the error bar represents the mean ± s.d. ($n = 3$). Statistical significance as calculated by two-tailed t-test is represented as *$p < 0.05$ and **$p < 0.01$.

Supplementary Figure 6

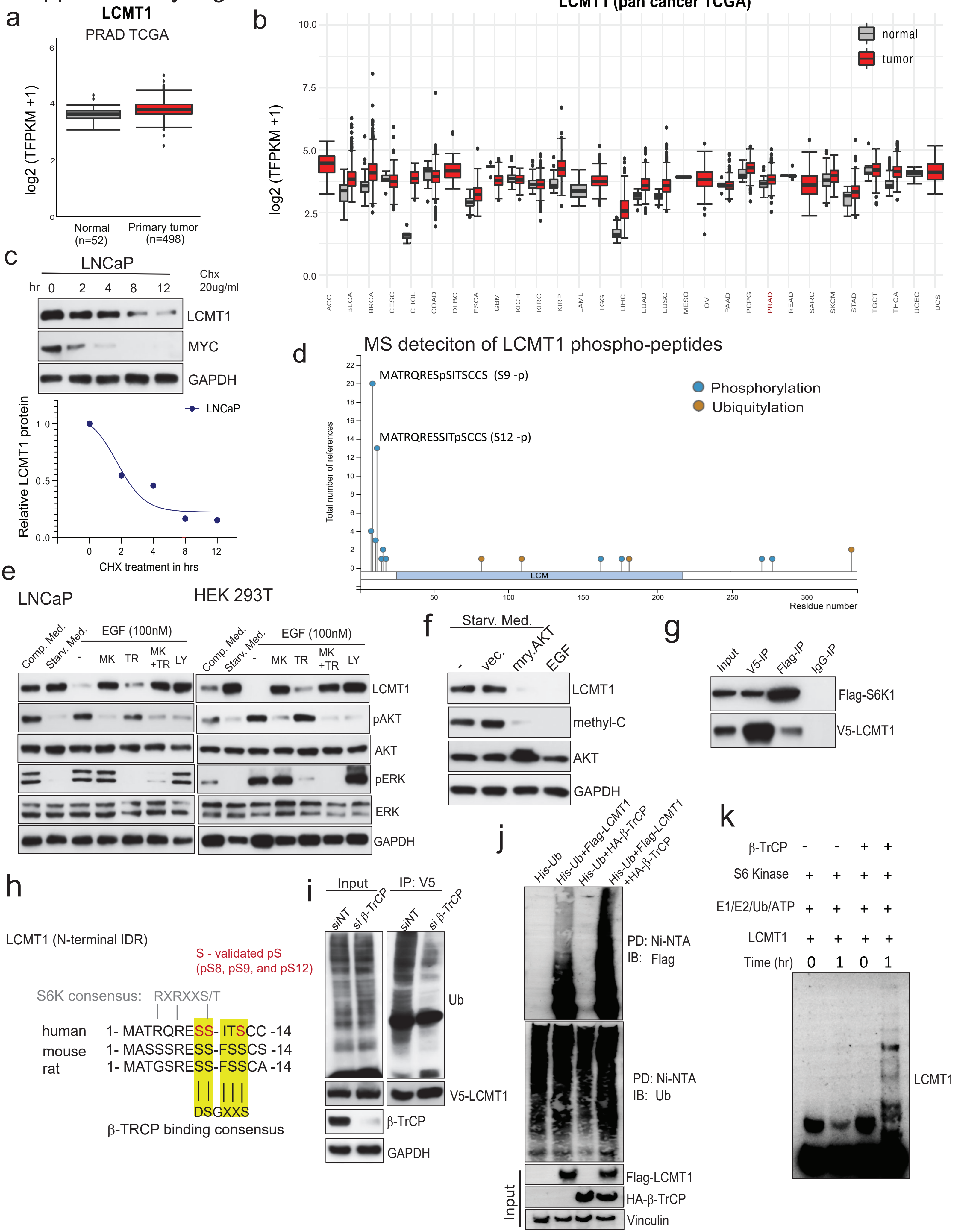

Supplementary Figure 6: LCMT1 transcript is not altered in prostate cancer. a, The box plot shows the normalized FPKM values of LCMT1 between Normal (n = 52) and Primary Tumor (n = 498) samples from The Cancer Genome Atlas – Prostate Adenocarcinoma (https://portal.gdc.cancer.gov/projects/TCGA-PRAD) Project. No significant change in LCMT1 RNA expression was observed. The middle line shows the median, the box limits show the 75th and 25th percentiles and the whiskers show the minimum and maximum values. **b,** The box plot shows the normalized FPKM values of LCMT1 in various primary tumor samples from the Cancer Genome Atlas Project. Boxplots with standard errors are shown. The middle line shows the median, the box limits show the 75th and 25th percentiles and the whiskers show the minimum and maximum values. **c,** LCMT1 is regulated at the translational/post-translational level. *Top*, immunoblot analysis of LCMT1 and MYC in LNCaP cells treated with 20μg/mL cycloheximide (Chx – translation inhibitor) for the indicated time points. MYC, a rapidly translated and highly labile protein, was used as a control for cycloheximide treatment. GAPDH served as a loading control. *Bottom*, the graph showing relative band densitometric values determined using ImageJ. **d,** Lollipop plot of high throughput (HTP) mass-spectrometry data sets displaying Serine-8, 9, and 12 (S8,9,12) as the most frequently phosphorylated residue in LCMT1 (https://www.phosphosite.org). The horizontal axis displays protein residues and domains, while the vertical axis reflects the number of papers reporting specified phosphorylation/ubiquitylation. **e,** Immunoblot panel revealing the effect of various PI3K/AKT/mTOR pathways or MEK inhibitors on LCMT1 expression. LNCaP and HEK293-T cells were grown in their respective complete medium or serum-starved (24h) or after starvation stimulated with EGF (6 h) with or without 1 h prior treatment with the indicated inhibitors followed by protein extraction and immunoblotting for the indicated proteins. **f,** Constitutively active mry.AKT blocks the restoration of LCMT1 upon serum starvation. HEK293-T cells were serum starved for 24h and either transfected with an empty vector or mry.AKT plasmid for 72h or activated with EGF (24h) followed by protein extraction and immunoblotting. **g**, S6K1 physically interacts with LCMT1 for phosphorylation. HEK293-T cells were co-transfected with V5-LCMT1 and Flag-S6K1 constructs followed by co-IP with V5 and Flag antibody. **h,** Position, and cross-species (human, mice, and rat) sequence conservation is shown for β-TRCP binding DSGXXS consensus, where X represents any possible residue, and D could be replaced by phospho-Serine. **i**, β-TrCP knockdown results in reduced LCMT1 polyubiquitinated levels. HEK293T cells were cotransfected with V5-LCMT1 plasmid with siNT or siβ-TrCP. 72h post-transfection, total protein lysates were prepared and used of co-IP with V5 antibody followed by immunoblotting with ubiquitin antibody. **j**, Increased polyubiquitination of LCMT1 upon β-TrCP overexpression *in vivo*. HEK293-T cells were transfected with His-ubiquitin along with Flag-LCMT1 or HA- β-TrCP or both followed by pull-down of ubiquitinated proteins using Ni-NTA beads and subsequent immunoblotting with Flag antibody. **k**, β-TrCP directly regulates LCMT1 ubiquitination. Ubiquitination of LCMT1 was reconstituted *in vitro* using purified E1/E2/Ub, ATP, S6K1, and unlabeled in vitro transcribed/translated LCMT1 and/or β-TrCP1. The completed reaction mix was then analyzed by immunoblotting with an anti-LCMT1 antibody. The data shown in c, e-g, and i-k, are representative of at least two independent biological replicates.

Supplementary Figure 7

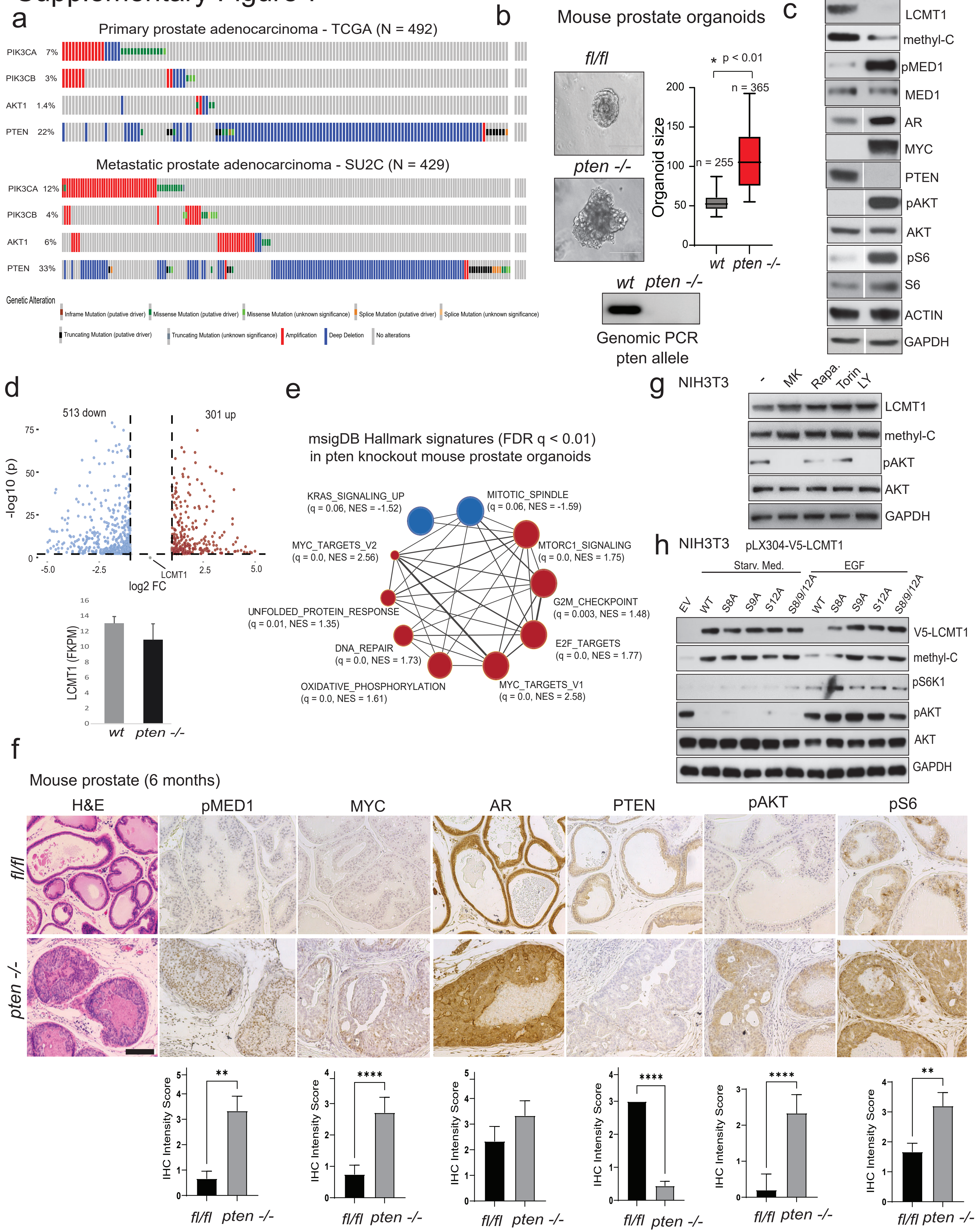

Supplementary Figure 7: *Pten* deletion leads to AKT/mTOR mediated LCMT1 loss/reduction in mouse prostate organoids. a, Aberrations in the Pten-PI3K-AKT pathway found in human primary and metastatic prostate cancer. Oncoprint from CBioPortal showing the frequency of deletions, mutations, and copy number alterations in PI3K pathway components, in the primary prostate cancer TCGA dataset, and metastatic prostate cancer SU2C dataset. **b,** Left: Phase-contrast image showing mouse prostate organoids developed from C57BL/6J (*Pten* wt) or B6.129S4-Ptentm1Hwu/J (*Pten* null) mice; Right: Graph showing the organoid size in *Pten* wt (n = 35) versus *Pten* null (n = 30) condition; Bottom: *Pten* allele deletion confirmation by genomic PCR. The middle line in the boxplot shows the median, the box limits show the 75th and 25th percentiles and the whiskers show the minimum and maximum values. Student's two-tailed t-test *$p < 0.05$. **c,** *Pten* deletion leads to LCMT1 loss/reduction with a reciprocal increase in p-MED1, AR, and MYC. Proteins extracted from the *Pten* wt and *Pten* null organoids were subjected to immunoblotting for the indicated proteins. Actin and GAPDH were used as the loading control. Note - spliced lanes for a few of the targets are from the same blot. **d,** Top: RNA-seq Volcano plot of differentially expressed genes in *Pten* null compared to *Pten* wt control organoids. p value < 0.05, using Wald test compared with control. |log2 fold change| >= 1. Bottom: Bar graph showing the LCMT1 expression. The error bar shows the s.d. among triplicates. **e,** *Pten* loss leads to hyper-activated MTORC1 and MYC signaling. GSEA network plot showing positive and negative enrichment in msigDB Hallmark signatures upon *Pten* deletion. **f,** Top, *Pten* loss is associated with increased p-MED1. Representative immunohistochemistry (IHC) and H&E staining images of the indicated proteins in *Pten* wt (treated as Normal) and *Pten* null (treated as HGPIN) mouse prostate. Scale bar represent 100μm applies to all panels. Bottom, bar graphs represent the IHC intensity scores. The data for quantification were acquired from n=3-5 animals per group. At least 5 images were evaluated per tissue. The error bar represents the s.d. among the replicates. Statistical significance as calculated by two-tailed t-test is represented as **$p < 0.01$ and ****$p < 0.0001$. **g,** Stabilization of LCMT1 upon inhibition of PI3K/AKT/mTOR pathway in mouse fibroblast cells. NIH-3T3 cells were treated with the indicated compounds for 24h, followed by protein extraction and immunoblotting for the indicated proteins. The data is representative of three independent biological replicates. **h,** Resistance to mitogen-induced degradation by S8,9,12A LCMT1 mutants in mouse fibroblast cells. NIH-3T3 cells were transfected with the empty vector (EV), V5-tagged wildtype (WT) LCMT1 or indicated mutant constructs. Twenty-four hours post-transfection, the cells were serum-starved for 24h and then either stimulated with the 100nM EGF or continued to grow in the absence of serum for another 24h. Protein extracts were analyzed by immunoblotting with antibodies for the indicated proteins. The data is representative of at least two independent biological replicates.

Supplementary Figure 8

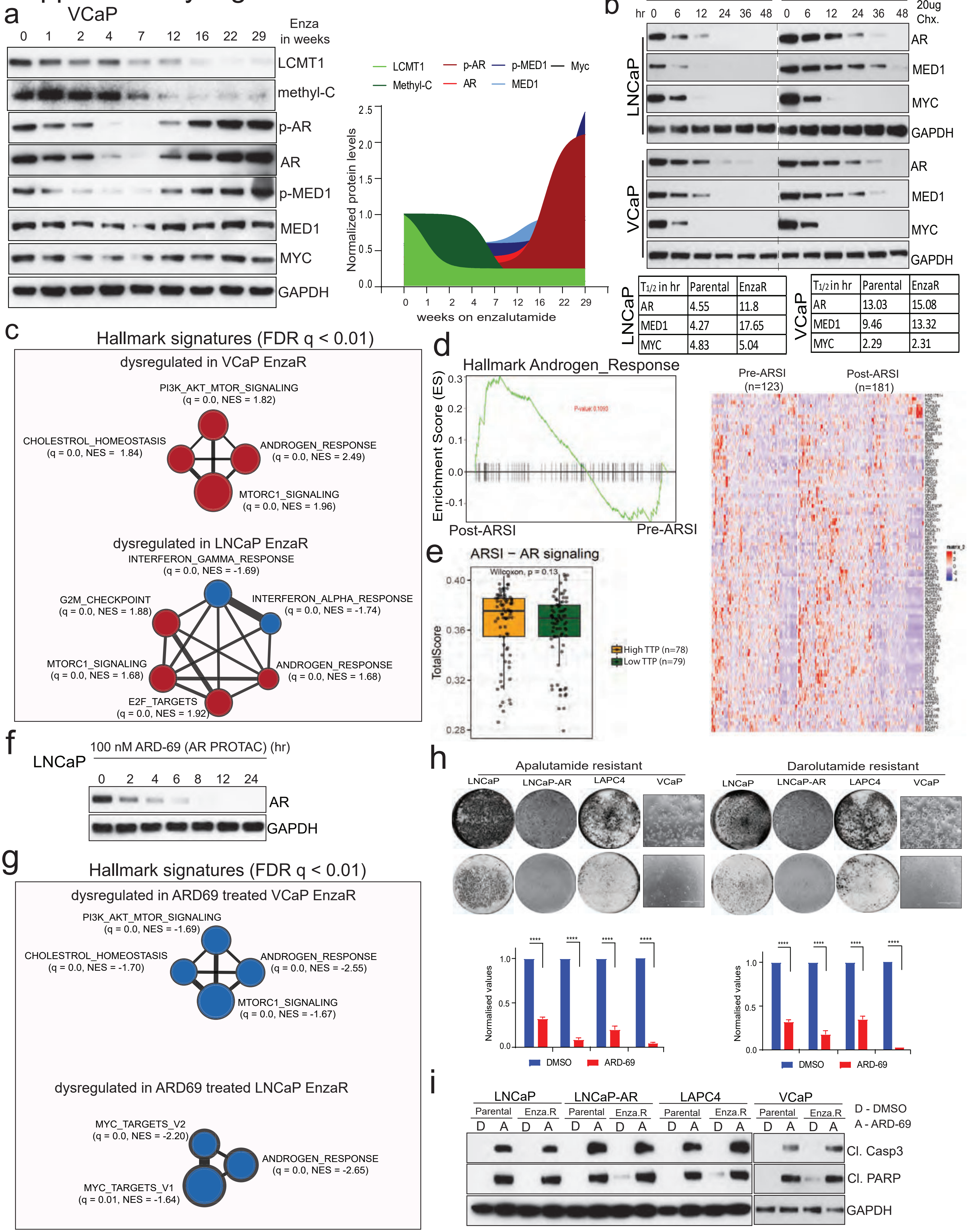

LNCaP

T1/2 in hr	Parental	EnzaR
AR	4.55	11.8
MED1	4.27	17.65
MYC	4.83	5.04

VCaP

T1/2 in hr	Parental	EnzaR
AR	13.03	15.08
MED1	9.46	13.32
MYC	2.29	2.31

Supplementary Figure 8: Continued AR-addiction in anti-androgen refractory prostate cancer cells. a, Evolution of enzalutamide refractory (EnzaR) state is associated with gradual loss of LCMT1 and Leucine-309 methylation. VCaP cells were grown in the presence of enzalutamide for 29 weeks. The cells were harvested at the indicated weeks, and the lysates prepared were subjected to immunoblotting for the indicated proteins. Normalized densitometric (ImageJ) plot for the levels of different proteins during the development of enzalutamide refractory state is shown. The experiment was performed independently in two different PCa cell lines. **b,** Increased AR-MED1 stability in EnzaR cells. Immunoblot showing AR and MED1 in the parental and enzalutamide-resistant cells treated with 20μg/mL Chx. MYC, a rapidly translated and highly labile protein, was used as a control for cycloheximide treatment. GAPDH served as a loading control. Table below show the calculated half-life of each protein between parental and EnzaR cells. The data is representative of n=3 biological replicates. **c,** GSEA network plots showing the significantly dysregulated msigDB Hallmark signatures in VCaP- and LNCaP-Enza-resistant cells (EnzaR) compared to their parental line. **d,** GSEA plot and heatmap for msigDB Hallmark Androgen_Response genes in pre- and post-ARSI samples. **e,** Box plot showing gene signature scores for msigDB Hallmark Androgen Response signature in samples post-ARSI therapy stratified by time to progression. For **d** and **e** the p values as shown were computed by Wilcoxon test. The centre line shows the median, the box limits show the 75th and 25th percentiles and the whiskers show the minimum and maximum values. **f,** ARD-69 – a potent AR degrader. LNCaP cells were treated with 100 nM ARD-69 for the indicated time points, and the lysates prepared were subjected to immunoblotting for AR. The data is representative of n=3 biological replicates. **g,** GSEA network plots showing significantly dysregulated msigDB Hallmark signatures in VCaP- and LNCaP-Enza-resistant cells (EnzaR) treated with 100nM ARD-69 for 12h compared to DMSO controls. FDR $q < 0.01$. NES: normalized enrichment score; FDR: False Discovery Rate. **h,** AR degradation affects the colony formation ability of apalutamide and darolutamide resistant PCa cells. Indicated cell lines were cultured either in the presence of vehicle control or 100nM ARD-69 for 12-14 days, followed by crystal violet staining (n=3). In the case of VCaP -representative bright field images are shown. Normalized quantification of crystal violet stain/cell viability is shown. The error bar represents the mean ± s.d. (n = 3), and statistical significance as calculated by two-tailed t-test is represented as ****$p < 0.0001$. **i,** Apoptosis induction upon AR degradation. The indicated parental cell lines or EnzaR derivatives were treated with either 100nM ARD-69 or DMSO for 48h. The data is representative of n=3 biological replicates. Lysates prepared from the cells were subjected to immunoblotting with antibodies against cleaved caspase 3 and cleaved PARP. GAPDH was used as a loading control.

Supplementary Figure 9

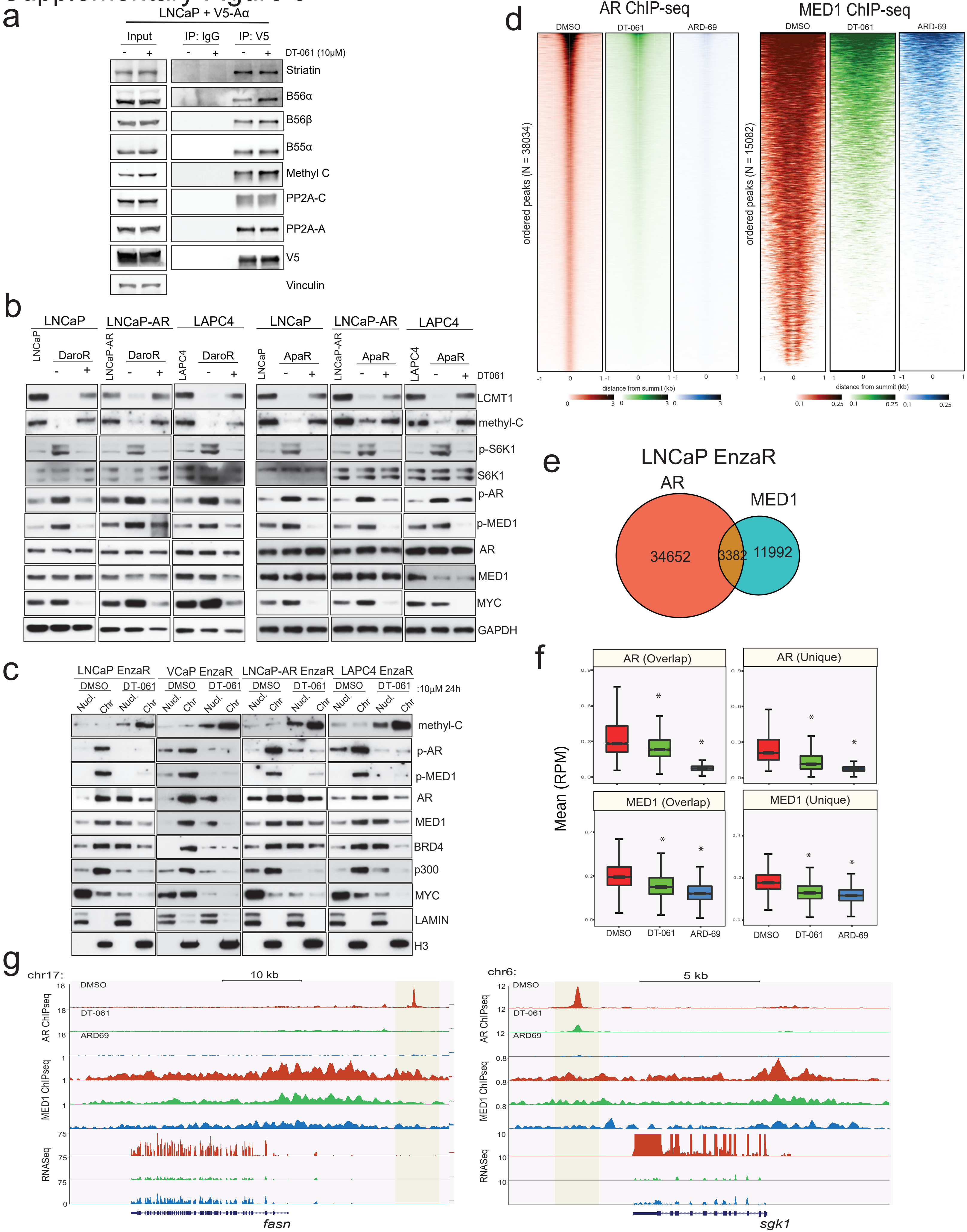

Supplementary Figure 9: DT-061 treatment restores LCMT1 resulting in reduced AR-MED1 from the chromatin in anti-androgen refractory PCa cells. a, DT-061 increases B56α containing PP2A heterotrimers in PCa cells. LNCaP cells stably expressing V5-PP2A-Aα were treated with vehicle or 10μM DT-061 for 12h. STRN was used as methylation insensitive controls and IgG was used as a negative IP control. Total lysate was used as input control. The data is representative of at least n=3 biological replicates. **b,** DT-061 restores LCMT1 protein and reduces p-AR and p-MED1 levels. Lysates prepared from the indicated parental and anti-androgen-refractory cell lines treated with either vehicle or 10 μM DT-061 for 24h were immunoprobed for the given proteins. MYC served as a positive control for the DT-061 treatment. GAPDH was used as the loading control. The data is representative of n=2 biological replicates. **c,** DT-061 treatment increases chromatin-bound methyl-C with a reciprocal decrease in p-AR and p-MED1**.** Chromatin and nuclear fractions from parental and EnzaR cells treated with either DMSO or 10μM DT-061 for 12h were used to probe the indicated proteins. BRD4 was used as an additional AR-cofactor and MYC as a positive control for DT-061 treatment. LAMIN and H3 served as controls for nuclear and chromatin fractions, respectively. The data is representative of n=2 biological replicates. **d,** Heatmap representation of genome-wide AR and MED1 ChIP-seq enrichment peaks in LNCaP EnzaR cells treated with vehicle or 10 μM DT-061 for 24 h. 500 nM ARD-69 treatment for 12h was used as a control to degrade AR from the chromatin. The enriched peaks in DMSO controls were rank-ordered based on their density and was used as the reference to assess AR and MED1 levels in all three conditions. AR and MED1 density are shown for the 1 kb region flanking the peak summit. **e,** Venn diagram showing the degree of overlap between AR and MED1 bound regions in LNCaP-EnzaR cells. **f,** DT-061 treatment leads to the eviction of AR and MED1 from chromatin. Boxplots of AR and MED1 enrichment (Reads per million mapped reads) at overlapping and unique regions. Note the higher AR and MED1 levels on the overlapping regions -indicating co-recruitment compared to their unique peaks that are reduced upon DT-061 treatment; standard error is shown (* indicates $p<0.0001$). As expected, ARD-69 demonstrated the highest loss of AR and MED1 at the overlapping and unique sites. The p values were computed by Wilcoxon test. The middle line shows the median, the box limits show the 75th and 25th percentiles and the whiskers show the minimum and maximum values. **g,** Genome browser tracks of AR and MED1 binding at *fasn* and *sgk1* loci in the indicated condition for LNCaP-EnzaR cells. The tracks at the bottom show the transcript levels of the corresponding loci.

Supplementary Figure 10

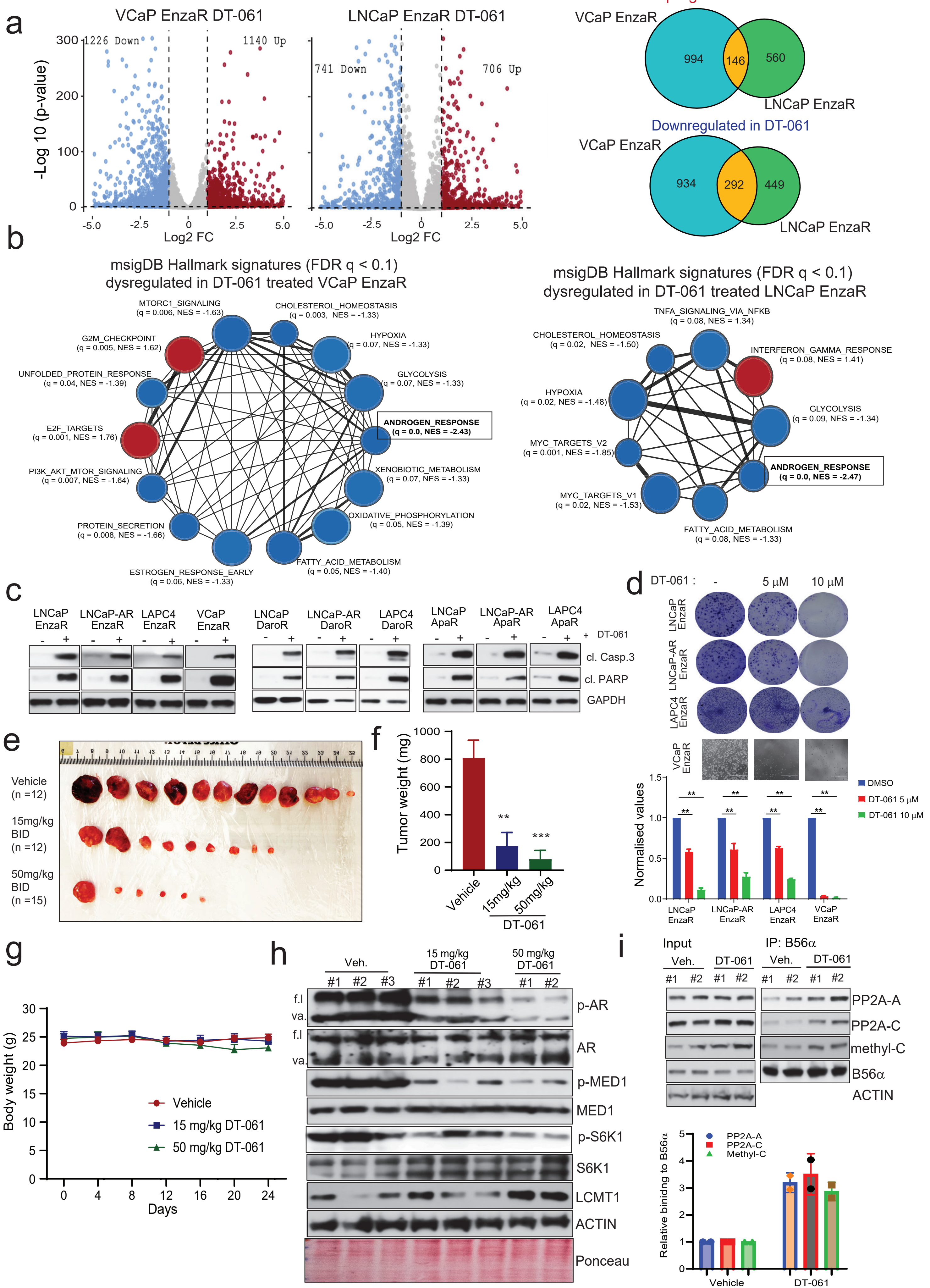

Supplementary Figure 10: DT-061 treatment blocks AR-mediated transcription and increases apoptosis-mediated cell death in anti-androgen refractory PCa cells. a, *Left*, Volcano plot showing differentially expressed genes in EnzaR cells treated with 10 μM DT-061 for 24h. *Right*, Venn diagram displaying up and down-regulated genes upon DT-061 treatment. **b,** GSEA network plots showing the significantly dysregulated msigDB Hallmark signatures in VCaP- and LNCaP-Enza-resistant cells (EnzaR) treated with DT-061 compared to DMSO control. The highest ranked negatively enriched Hallmark Androgen_Response signature is highlighted with a box. GSEA with FDR $q < 0.1$. NES: normalized enrichment score; FDR: False Discovery Rate. **c,** DT-061 induces apoptosis in anti-androgen refractory cells. The indicated anti-androgen refractory cells were treated with 10μM DT-061 or DMSO for 24h. Lysates prepared from the cells were subjected to immunoblotting with antibodies against cleaved caspase 3 and cleaved PARP. GAPDH was used as a loading control. The data is representative of n=2 biological replicates. **d,** DT-061 inhibits the colony formation ability of EnzaR cells. Indicated EnzaR cells were cultured in the absence or presence of the 5 and 10 μM DT-061 for 12days, followed by crystal violet staining (n=3). In the case of VCaP -representative bright field images are shown. Quantification of the colony formation assay is shown where the error bar represents the mean ± s.d. (n = 3). Statistical significance calculated by two-tailed t-test is represented as ** $p < 0.01$. **e,** DT-061 blocks enzalutamide refractory prostate cancer growth *in vivo*. Dissected tumors from indicated treatment groups. **f,** Average tumor weight from different treatment groups, as in e, is shown. The error bar represents the mean ± s.d. among the replicates. Statistical significance as calculated by two-tailed t-test is represented as **$p < 0.01$ and ***$p< 0.001$. **g,** Mean body weight ± s.d. in VCaP EnzaR xenograft shows that DT-061 treatment does not affect the animal weight. **h**, DT-061 mediated stabilization of LCMT1 is accompanied by decreased phosphorylation of AR and MED1 *in vivo*. Lysates from the tumor tissues harvested from the animals treated with vehicle (n=3), 15 mg/kg (n=3), and 50 mg/kg (n=2) of DT-061 were subjected to immunoblotting with the indicated antibodies. Ponceau stain was used as loading control. **i**, DT-061 increases the AB56αCme heterotrimers *in vivo.* Tumor lysates used for CoIP with B56α followed by immunoblotting with the indicated antibodies. Bar graphs show relative quantification of PP2A-A, PP2A-C, PP2A-Cme with B56α between vehicle (n = 2) and DT-061 (n = 2) treated group. Standard error represents the mean ± s.d. of the biological replicates.
