# Supplementary material for: Loss of LCMT1 and biased protein phosphatase 2A heterotrimerization drive prostate cancer progression and therapy resistance
Source: Nat Commun. 2023 Aug 29;14:5253. doi: 10.1038/s41467-023-40760-6 (PMC10465527; doi:10.1038/s41467-023-40760-6)
Supplement: Supplementary file 3 — Description of Additional Supplementary Files [file 41467_2023_40760_MOESM3_ESM.pdf]

Description of Additional Supplementary Files

Supplementary Data 1. The excel sheet contains information on antibodies, mouse strains, cell lines, sgRNA sequence, plasmid constructs, drugs, reagents kits, primers, siRNA, and software used in the study.
